# Mitochondrial citrate accumulation drives alveolar epithelial cell necroptosis in lipopolysaccharide-induced acute lung injury

**DOI:** 10.1038/s12276-022-00889-8

**Published:** 2022-11-28

**Authors:** Hui-Hui Yang, Hui-Ling Jiang, Jia-Hao Tao, Chen-Yu Zhang, Jian-Bing Xiong, Jin-Tong Yang, Yu-Biao Liu, Wen-Jing Zhong, Xin-Xin Guan, Jia-Xi Duan, Yan-Feng Zhang, Shao-Kun Liu, Jian-Xin Jiang, Yong Zhou, Cha-Xiang Guan

**Affiliations:** 1 Department of Physiology, School of Basic Medical Science, Central South University, Changsha, Hunan China; 2 Department of Geriatrics, Respiratory Medicine, Xiangya Hospital, Central South University, Changsha, Hunan China; 3 Department of Cardiovascular Surgery, Xiangya Hospital, Central South University, Changsha, Hunan China; 4 Department of Pulmonary and Critical Care Medicine, the Second Xiangya Hospital, Central South University, Changsha, Hunan China; 5 State Key Laboratory of Trauma, Burns, and Combined Injury, Department of Trauma Medical Center, Daping Hospital, Army Medical University, Chongqing, China

**Keywords:** Infection, Respiratory tract diseases

## Abstract

Necroptosis is the major cause of death in alveolar epithelial cells (AECs) during acute lung injury (ALI). Here, we report a previously unrecognized mechanism for necroptosis. We found an accumulation of mitochondrial citrate (citrate^mt^) in lipopolysaccharide (LPS)-treated AECs because of the downregulation of Idh3α and citrate carrier (CIC, also known as Slc25a1). shRNA- or inhibitor–mediated inhibition of Idh3α and Slc25a1 induced citrate^mt^ accumulation and necroptosis in vitro. Mice with AEC-specific Idh3α and Slc25a1 deficiency exhibited exacerbated lung injury and AEC necroptosis. Interestingly, the overexpression of Idh3α and Slc25a1 decreased citrate^mt^ levels and rescued AECs from necroptosis. Mechanistically, citrate^mt^ accumulation induced mitochondrial fission and excessive mitophagy in AECs. Furthermore, citrate^mt^ directly interacted with FUN14 domain-containing protein 1 (FUNDC1) and promoted the interaction of FUNDC1 with dynamin-related protein 1 (DRP1), leading to excessive mitophagy-mediated necroptosis and thereby initiating and promoting ALI. Importantly, necroptosis induced by citrate^mt^ accumulation was inhibited in FUNDC1-knockout AECs. We show that citrate^mt^ accumulation is a novel target for protection against ALI involving necroptosis.

## Acute Lung injury: Citrate as a culprit

The process of inflammation-induced cell death, necroptosis, in acute lung injury can be driven by the excessive accumulation of the small biomolecule citrate (a key intermediate of the tricarboxylic acid (TCA) cycle) inside the energy-processing parts of cells called mitochondria. Hui-Hui Yang in Central South University, Hunan, China, and colleagues uncovered this previously unknown mechanism for necroptosis in mouse lung cells exposed to lipopolysaccharide molecules in order to model acute lung injury. They described details of the molecular interactions that allow citrate to damage mitochondria and initiate cell death. They identified a molecular mechanism that could reduce the accumulation of citrate and protect the lung cells from necroptosis. The results suggest that drug treatments to reduce citrate accumulation could offer a novel route to protecting against forms of acute lung injury that involve necroptosis.

## Introduction

Acute lung injury (ALI) and its most severe form, acute respiratory distress syndrome (ARDS), are life-threatening conditions. These disease conditions are initiated by widespread diffuse damage to alveolar epithelial cells (AECs) and uncontrolled inflammation, which is characterized by bilateral chest radiographical opacities with severe hypoxemia^1,2^. Although substantial progress has been made in understanding the epidemiology and biology of ARDS over the past 5 decades, the pathogenic mechanisms of ALI/ARDS have not been fully identified. In fact, there is no proven pharmacological treatment for ARDS. Given that the ARDS fatality rate is 40%^3^, it is critical to explore the molecular mechanisms underlying ARDS pathogenesis.

AECs are essential for maintaining the structural and functional integrity of the lung^4^. Diffuse AEC injury is critical in the occurrence and development of ALI/ARDS^5^. Direct or indirect injury factors attack and destroy the alveolar structure, which activates macrophages and neutrophils by releasing damage-associated molecular patterns (DAMPs), rapidly amplifying the inflammatory response and exacerbating ALI/ARDS^4,6–8^. Recent studies have indicated that necroptosis but not apoptosis is the dominant type of AEC death in lipopolysaccharide (LPS)-induced ALI^9^. Necroptosis, which was first reported in 2005^10^, is a form of regulated cell death triggered by disturbances in intracellular and extracellular homeostasis. Necroptosis requires receptor-interacting protein kinase 3 (RIPK3) and is mediated by mixed lineage kinase domain-like pseudokinase (MLKL)^11^. RIPK3 phosphorylates MLKL. Then, phosphorylated MLKL oligomerizes and translocates to the cell membrane to form plasma membrane pores that cause the cell to swell in the presence of injury factors such as LPS^11,12^. We have reported that inhibition of AEC necroptosis attenuates the LPS-induced ALI in mice^13,14^. However, the molecular mechanism of AEC necroptosis in LPS-induced ALI is not fully understood.

Metabolic reprogramming is a rapidly growing topic in the field of inflammation research^15^. Cells reprogram glucose metabolism by transitioning from oxidative phosphorylation to glycolysis even in the presence of oxygen because glycolysis provides rapidly available energy and an ample supply of precursors for macromolecule biosynthesis^16^. Moreover, glucose metabolites, which are signaling molecules, have important functions in regulating inflammation and immune responses^17^. Citrate is produced in the mitochondrial tricarboxylic acid (TCA) cycle *via* citrate synthase-mediated conversion of oxaloacetate and acetyl-CoA. Mitochondrial citrate (citrate^mt^) is normally converted to isocitrate, which is decarboxylated by isocitrate dehydrogenase (IDH3) to generate α-ketoglutarate (αKG). In addition, citrate can be transported from the mitochondria into the cytosol (citrate^cy^) *via* the citrate carrier (CIC, also known as Slc25a1). Hence, the expression of IDH3 and CIC is highly correlated with citrate^mt^ homeostasis. Citrate^cy^ can be metabolized to produce acetyl-CoA and oxaloacetate *via* cytosolic ATP citrate lyase (Acly)^18–20^. Acetyl-CoA provides material for the phospholipid precursors of arachidonic acid, which can produce prostaglandin proinflammatory mediators *via* cyclooxygenase 2^21^. Therefore, citrate^cy^ is an important signaling molecule. However, the role of citrate^mt^ in AECs in LPS-induced ALI has never been investigated.

Mitochondrial health, which is maintained by mitochondrial quality control (MQC), is essential for the survival and function of eukaryotic organisms. MQC is a complex signaling response that affects mitochondrial biogenesis, fusion/fission, mitophagy, and the mitochondrial unfolded protein response^22,23^. Dynamin-related protein 1 (DRP1), which is a member of the dynamin family of GTPases, is a major regulator of mitochondrial fission^24^. FUN14 domain-containing protein 1 (FUNDC1) is a novel mitochondrial membrane-associated protein that recruits DRP1 to drive mitochondrial fission in response to hypoxic stress^25^. Strikingly, the phosphorylation of DRP1 at serine 616 (Ser^616^) has a crucial role in the regulation of DRP1 activity. Ser^616^ phosphorylation promotes DRP1 recruitment to mitochondria and subsequent mitochondrial fission^26^. Moreover, mitochondrial fragmentation is required for mitophagy^27^. Mitophagy is a “self-eating” process, while, excessive mitophagy is maladaptive and linked to cell death^28,29^. DRP1-mediated mitochondrial fission contributes to excessive mitophagy^30^, which significantly reduces mitochondrial mass and causes ATP exhaustion, resulting in stress-induced death *via* necroptosis^29^. For example, the interaction of DRP1 with retinoblastoma protein mediates necroptosis and hepatic injury induced by cadmium exposure^31^. Thus, mitochondrial dysfunction, such as DRP1-induced excessive mitophagy, triggers necroptosis.

Recently, we showed that extracellular citrate could act as a DAMP to activate macrophages and promote LPS-induced ALI in mice^32^. However, the role of citrate^mt^ in the early stage of ALI remains unknown. A previous study reported that citrate concentrations were positively associated with mitochondrial dysfunction in nonalcoholic fatty liver disease^33^. Thus, it is plausible that there is a link between citrate^mt^ and mitochondrial dysfunction. In addition, citrate has been shown to inhibit polynucleotide phosphorylase (PNPase) activity by binding to the active site of two distantly related PNPases^34^. Taken together, these exciting findings prompted us to hypothesize that excessive citrate^mt^ might bind to FUNDC1 and consequently initiate the recruitment of DRP1 to drive mitochondrial fission, leading to excessive mitophagy and triggering necroptosis in AECs during ALI.

In this study, we first examined citrate^mt^ accumulation in AECs that were challenged with LPS ex vivo and in vivo. The overexpression of Idh3α and Slc25a1 decreased citrate^mt^ levels and rescued AECs from LPS-induced necroptosis. Conversely, inhibiting Idh3α and Slc25a1 with shRNA or inhibitors induced citrate^mt^ accumulation and necroptosis. Mechanistically, we found that citrate^mt^, which directly binds to FUNDC1, recruited cytoplasmic DRP1 to mitochondria, which exacerbated mitochondrial fission, resulting in excessive mitophagy that triggered necroptosis in AECs during the development of ALI/ARDS.

## Materials and methods

### Animals

C57BL/6J mice (male, 8 weeks old, purchased from Hunan SJA Laboratory Animal Co., Ltd) were bred and housed in the pathogen-free facility of Central South University at 25 °C with a 12:12 h light:dark cycle. The mice had ad libitum access to food and water. All mice underwent routine health status checks by a certified veterinarian. The animals were randomly assigned to the experimental and control groups.

### Animal treatments

To induce the ALI model, the mice were intratracheally administered LPS (Sigma‒Aldrich, from *Escherichia coli O111: B4*, USA, 5 mg/kg body weight) as previously described^35^. Twelve hours after LPS administration, the mice were sacrificed. To investigate the effects of IDH3 and CIC inhibition on the severity of ALI, we used TBT (Aladdin, an IDH3 inhibitor, 20 mg/kg body weight) and BTA (Sigma‒Aldrich, a CIC inhibitor, 20 mg/kg body weight). The inhibitors were simultaneously injected intraperitoneally every day for three days. Then, the mice were intratracheally administered LPS (2.5 mg/kg) for 12 h. Specific adenovirus-mediated shRNAs (1 × 10^8^ PFU/20 g) targeting Idh3α or Slc25a1 in AECs were intratracheally injected 10 days before LPS (2.5 mg/kg) treatment.

### Bronchoalveolar lavage fluid (BALF) collection

BALF was collected as previously described^35^. Briefly, the lungs were lavaged with 0.8 mL of ice-cold PBS three times. The recovered fluid was centrifuged at 1500 rpm for 5 min at 4 °C, and then the supernatant was collected to measure the citrate concentration.

### Histopathology and inflammation scores

Histopathological analysis of paraffin-embedded lung tissue was performed on lung sections stained with H&E by standard procedures. The severity of morphological changes (neutrophils in the alveolar space, hemorrhage, hyaline membranes, pertinacious debris filling the airspaces, and septal thickening) was assessed semiquantitatively using a numeric inflammation score. The mean score was considered the inflammation score (0–4) and was determined by three independent pathologists.

### Untargeted metabolomics assay

Untargeted metabolomics assays were performed as previously described^36^. Briefly, untargeted metabolomics assays for glucose were performed by gas chromatography‒mass spectrometry (Shanghai Profleader Biotech Co., Ltd.). Metabolomics instrumental analysis was performed on an Agilent 7890 A gas chromatography system coupled to an Agilent 5975 C inert MSD system (Agilent Technologies Inc., CA, USA), and twenty-three untargeted metabolites were analyzed.

### ELISA

Serum levels of TNF-α and IL-1β were measured with ELISA kits (Invitrogen, TNF-α: # T88-7324; IL-1β: 88–7013) according to the manufacturer’s instructions. The levels were determined by comparing the optical density (450 nm and 570 nm) against a standard curve.

### Cell culture

Mouse lung AECs (MLE12 cells, ATCC CRL-2110) were maintained in HITES medium (DMEM/F12 medium containing hydrocortisone, insulin, transferrin, estradiol, and selenium) supplemented with 2% fetal bovine serum (Gibco, USA) and 1% (v/v) penicillin/streptomycin (Solarbio) at 37 °C and 5% CO_2_.

### Cell treatments

Cells were treated with 0, 1, 3.3, and 10 μg/mL LPS for 3 h, 12 h, and 24 h. Cells were treated simultaneously with the IDH3 inhibitor (TBT, 100 nM) and CIC inhibitor (BTA, 2 mM) for 3 h and 24 h.

### Lentivirus-mediated knockdown (or overexpression) of Idh3α and Slc25a1

To manipulate citrate^mt^ levels in MLE12 cells, packed empty LV vectors (TTCTCCGAACGTGTCACGT), LV-mediated sh-Idh3α vectors (cgGAGAACTGTAAAGACATTA; GenBank accession number NM_029573), and LV-mediated sh-Slc25a1 vectors (gtATTCATCATCTACGATGAA; GenBank accession number NM_153150) were designed and synthesized by GeneChem (Shanghai, China). MLE12 cells were simultaneously transfected with the shIdh3α lentivirus (MOI: 50), shSlc25a1 lentivirus (MOI: 50) and negative control (shCtrl lentivirus, MOI: 50) in the presence of 1×HitransG P (Lot: REVG005, GeneChem). For overexpression experiments, lentiviruses for mouse Slc25a1 (NM_153150) and Idh3α (NM_029573) were purchased (GeneChem), and lentiviral transduction of MLE12 cells was performed at an MOI of 25. The empty construct was used as the control for all experiments. The cells were washed with fresh complete medium 16 h later and then cultured for an additional 80 h. The efficiency of Idh3α and Slc25a1 knockdown (or overexpression) was assessed by immunoblotting.

### Generation of FUNDC1-KO cell lines by CRISPR/Cas9

To stably knock out the expression of FUNDC1 in MLE12 cells, a mouse FUNDC1-KO kit was purchased from cas9x^TM^ (GeneID: 72018, Cat.: KT317685). MLE12 cells were transfected with 1 μg of the FUNDC1 sgRNA plasmid plus 1 μg of the Cas9 plasmid according to the manufacturer’s recommendations. Following 2 μg/mL puromycin (GeneChem, REVG1001) selection for five days, the cells were sorted into 96-well plates. Single-cell clones were expanded and validated as FUNDC1 KO clones by immunoblotting.

### Flow cytometry

To measure the degree of apoptosis and necrosis following LPS treatment in vitro, cells were stained with Annexin V and propidium iodide (BD, USA) for 15 min at room temperature in the dark and then harvested with gentle scraping. The cells were then washed with PBS, and cytometry buffer was added. The cells were analyzed using a BD FACS Verse (BD Biosciences, San Jose, CA, USA) flow cytometer with an excitation wavelength of 488 nm and an emission wavelength of 530 nm and were analyzed using FlowJo software (version 10, Tree Star Inc., San Jose, CA, USA).

### Transmission electron microscopy (TEM)

MLE12 cells were fixed in 2.5% glutaraldehyde at 4 °C for 2 h and postfixed with 1.5% osmium tetroxide for 2 h. Then, the cell pellets were dehydrated with acetone and embedded in resin. The pieces were sectioned at a thickness of 70 nm and stained with lead citrate after hardening. Cellular ultrastructure was observed by TEM (FEI Tecnai 20, Hillsboro, OR, USA), as previously described^31^.

### Immunofluorescence analysis

Immunofluorescence analysis was performed as previously described^35^. Briefly, the cells were seeded on glass coverslips, treated as indicated, and fixed with 4% paraformaldehyde for 15 min at room temperature. Subsequently, the cells were washed three times with PBS and incubated with 1% BSA (Carl Roth) in PBS for 30 min at room temperature. The coverslips were incubated with diluted primary anti-MLKL or anti-TOM20 antibodies in blocking buffer in a humid chamber overnight at 4 °C. After being incubated, coverslips were washed with PBS three times and incubated with secondary antibodies for 1 h at room temperature. The cells were stained with 1× DAPI (Solarbio, China) for 2 min and washed three times with ddH_2_O. Imaging was performed with a Carl Zeiss LSM700 laser scanning confocal microscope (Prenzlauer, Berlin, Germany).

### Coimmunoprecipitation (Co-IP)

For FUNDC1 and DRP1 immunoprecipitation, cell extracts were prepared using IP lysis buffer (50 mM Tris-HCl at pH 7.4, 150 mM NaCl, 0.5% Nonidet P-40, 10% glycerol, and 1 mM EDTA) and fresh protease inhibitors. The lysates were precleared by centrifugation for 10 min. Ten percent of the supernatants were reserved for measuring protein input. The remaining lysate was incubated with IgG-coupled magnetic beads (Pierce™ Protein G Magnetic Beads) overnight at 4 °C on a rotator wheel. Following five washes with lysis buffer, the immune complex beads were heated at 100 °C in 1× loading buffer (75 mM Tris-HCl at pH 6.8, 10% glycerol, 2% SDS, 0.05% bromophenol blue, and 2.5% β-mercaptoethanol) prior to being loaded on 12% SDS-PAGE gels. After electrophoresis, the separated proteins were transferred to polyvinylidene fluoride (PVDF) membranes (Millipore, USA) in transfer buffer (25 mM Tris, 0.192 M glycine, and 20% methanol), and the transferred membranes were immunoblotted with antibodies.

### Mitochondrial membrane potential (MMP) assay

A mitochondrial-specific cationic dye (JC-1) was used for an enhanced MMP assay (kit from Beyotime, China). MLE12 cells were seeded in 24-well plates. After LPS or LV-mediated sh-Idh3α vector and LV-mediated sh-Slc25a1 vector treatment, JC-1 was added and incubated at 37 °C for 30 min, and the cells were washed three times with JC-1 buffer. Images were obtained using a Carl Zeiss LSM700 laser scanning confocal microscope.

### Cytoplasmic and mitochondrial extraction

Cells and lung tissue were collected by trypsin release (Sigma‒Aldrich) and centrifuged at 300 g at 4 °C for 5 min. Each time, 5 × 10^7^ cells were extracted. The cells (5 × 10^7^) were resuspended by adding 1.0 mL of precooled lysis buffer and grinding the cell suspension in an ice bath at 0 °C. The extracts were centrifuged at 1000 g at 4 °C for 5 min, and the supernatant was transferred to a new EP tube and recentrifuged. The pellet containing nuclei and unbroken cells was discarded, and the supernatant was centrifuged at 12,000 g at 4 °C for 10 min. The resulting supernatant contained cytoplasm, and the pellet contained mitochondria. After transferring the supernatant to a new EP tube, 0.5 mL of Wash Buffer was added to resuspend the mitochondrial pellet, which was centrifuged at 1000 g at 4 °C for 5 min. The supernatant contained cytoplasm was centrifuged at 12,000 g at 4 °C for 10 min. The supernatant was discarded, and the precipitates were collected and dissolved in RIPA buffer solution (Solarbio, China).

### Gene expression analysis

Total RNA was harvested from cells and lung tissue using RNAiso Plus (TaKaRa, Japan) according to the manufacturer’s instructions. RNA integrity was assessed by agarose gel electrophoresis. The quality of the RNA preparation was based on the 28 S/18 S ribosomal RNA ratio and was measured spectrophotometrically (Thermo Fisher Scientific, USA). One microgram of total RNA was reverse-transcribed into cDNA in 20 μL of reaction volume using a PrimeScript™ RT Reagent Kit with gDNA Eraser (RR047A, TaKaRa, Japan). The primers used for gene expression analysis are listed in Supplementary Table 1. Real-time qPCR was performed using TB Green® Premix Ex Taq™ (Tli RNaseH Plus, RR420A, TaKaRa, Japan), and β-actin was used as a reference. The quantitative PCR conditions were as follows: 95 °C for 30 s, 40 cycles of 95 °C for 5 s, 60 °C for 30 s, and melting at 95 °C for 10 s, 65 °C for 5 s, 95 °C/0.5 °C. Relative gene expression was determined by the 2^−ΔΔCt^ method.

### Immunoblotting

Cells and lung tissue lysates were extracted with RIPA buffer (high) (Solarbio, R0010, China) containing 1% protease inhibitors (MCE, USA) and a phosphatase inhibitor cocktail. The samples were mixed with SDS lysis buffer (10% SDS, 0.25 M Tris-HCl at pH 6.8, 50% glycerol, 1% β-mercaptoethanol, and bromophenol blue) and separated by SDS‒PAGE. For nonreducing gel analysis, the cells were lysed in 20 mM Tris (pH 7.0) containing 0.5% NP40, 250 mM NaCl, 3 mM EDTA, 3 mM EGTA, 0.5 mM phenylmethylsulfonyl fluoride, 20 mM glycerol phosphate, 1 mM sodium vanadate, and 1 μg/mL leupeptin and separated by native PAGE. Then, the proteins were transferred to PVDF membranes (Millipore, USA). After being blocked with 5% skim milk or BSA for 1 h, the membranes were incubated with primary antibodies at 4 °C overnight and then with the HRP-labeled secondary antibodies for 1 h at room temperature. The antibodies used for protein expression analysis are listed in Supplementary Table 2. Bands were visualized with an enhanced chemiluminescence kit (WBLUR0500, Millipore, USA).

### Quantification and Statistical Analysis

Sample sizes (replicates, animals) are traceable as individual data points in each figure. Ex vivo experiments were repeated at least twice. Data involving animals are pooled data of at least two independent experiments. All tests used to determine statistical significance were two-sided. Statistical comparisons between two groups were determined by an unpaired *t* test. Differences among variables were assessed by ANOVA. A *P* value less than 0.05 was considered statistically significant. Details on specific statistical tests and the exact value of *n* are in each figure legend. All statistical analyses were performed using SPSS 22.0 (IBM, Chicago, IL) or GraphPad Prism 9 (San Diego, CA, USA). A molecular docking model of FUNDC1-citrate binding was performed using AutoDock and PyMol. The schematic was created with BioRender.com.

## Results

### Citrate accumulates in the lungs and MLE12 cells in response to LPS challenge

**Fig. 1:**
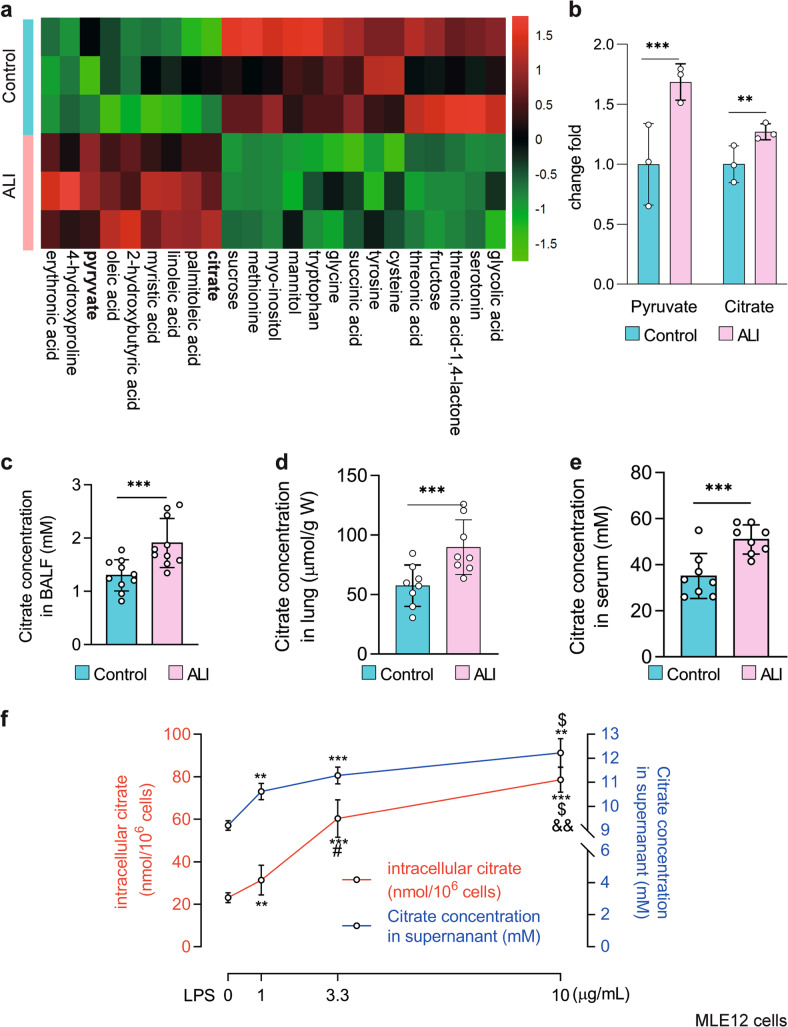
Citrate accumulates in the lungs and MLE12 cells challenged with LPS. **a** Heatmap showing increased levels of pyruvate and citrate in the lungs of mice treated with LPS compared to normal saline (*n* = 3). **b** The fold changes of pyruvate and citrate were calculated relative to the normal saline group (*n* = 3). Dots, individual mice. **c**–**e** The citrate concentrations in the BALF, lungs, and serum of LPS-induced ALI mice were increased (*n* = 8–10). **f** LPS-treated MLE12 cells (12 h) showed enrichment of citrate (*n* = 3). The data are shown as the mean ± SD. **P < 0.01 and ***P < 0.001.

We previously demonstrated that metabolic reprogramming occurs in the lungs after the induction of ALI^37^. To further investigate this observation, we employed a LPS-induced ALI mouse model (Supplementary Fig. 1a–c). Then, we used gas chromatography‒mass spectrometry (GC‒MS) and untargeted metabolomics analysis to measure the intermediate metabolites derived from glucose in the lungs of ALI mice and observed that pyruvate was increased in the lungs (Fig. 1a, b). Interestingly, citrate, the crucial substrate of the TCA cycle, was also elevated compared to that in control samples (Fig. 1a, b). In addition, citrate concentrations in the BALF, lungs, and serum of LPS-treated mice were higher than those in controls (*n* = 8–10, *P* < 0.001, Fig. 1c–e), which was consistent with the GC‒MS results. MLE12 cells were stimulated with LPS to mimic AECs injury (Supplementary Fig. 1d–f). Interesting, similar increases in citrate concentrations were observed in the cells and supernatants of MLE12 cells cultured in the presence of LPS, as shown in Fig. 1f. Taken together, these results indicate that citrate accumulates in the lungs and in MLE12 cells in response to LPS challenge.

### LPS impairs citrate metabolism and transport in vivo and in vitro

**Fig. 2:**
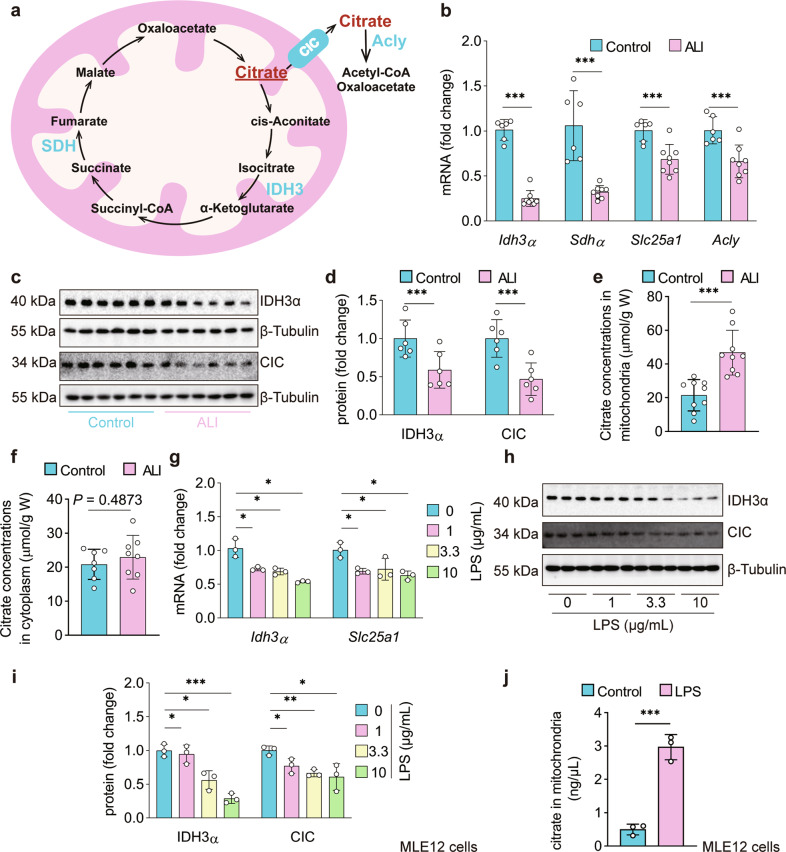
Impaired citrate metabolism and transport in the lungs of mice and MLE12 cells with LPS challenge. **a** The pathway of citrate metabolism and transport. **b** Expression of *Idh3α*, *Sdhα*, *Slc25a1*, and *Acly* mRNA in the lungs 12 h after LPS or normal saline treatment. The fold changes were calculated relative to the saline group (*n* = 6–8). **c**–**f** Representative immunoblots of lung tissue lysates 12 h after LPS treatment showing decreased protein levels of IDH3α and CIC (*n* = 6 mice per group) and increased citrate^mt^ but not citrate^cy^ in the lungs of ALI mice (*n* = 8–9 mice per group). **g**–**j** IDH3α and CIC mRNA and protein levels were downregulated, whereas increased citrate^mt^ was observed in LPS-treated MLE12 cells (*n* = 3). The data are shown as the mean ± SD. *P < 0.05, **P < 0.01, and ***P < 0.001.

Citrate^mt^ participates in the TCA cycle through the downstream key enzymes IDH3 and succinate dehydrogenase (SDH)^18^. Citrate^mt^ can be transferred to the cytoplasm by the CIC. Citrate^cy^ is converted to oxaloacetate and acetyl-CoA by ATP citrate lyase (Acly)^20^ (Fig. 2a). We found that *Idh3α*, *Sdhα*, *Slc25a1*, and *Acly* mRNA levels were markedly downregulated by 75.9%, 67.3%, 31.0%, and 33.2%, respectively, in the lungs after 12 h of LPS treatment (Fig. 2b), which indicates the accumulation of citrate^mt^ in the lungs and MLE12 cells in response to LPS challenge. The immunoblot results showed that the protein expression of IDH3α and CIC was decreased by 41.2% and 53.2%, respectively, in the lung tissue of LPS-induced ALI mice compared with the controls (Fig. 2c, d). To further confirm the alterations in citrate in the mitochondria and cytoplasm, we measured the concentrations of citrate^mt^ and citrate^cy^. The results revealed that the concentration of citrate^mt^ was significantly increased in the lung tissue of LPS-induced ALI mice (21.45964 ± 8.777 μmol/g) compared with the controls (46.795 ± 14.224 μmol/g) (Fig. 2e). In contrast, citrate^cy^ was not significantly different between the two groups (Fig. 2f). These results were consistent with our findings that the protein expression levels of IDH3α and CIC were suppressed. The gene and protein expression levels of Idh3α and CIC were also inhibited in LPS-treated MLE12 cells (Fig. 2g–i), and citrate^mt^ was significantly increased (Fig. 2j). Taken together, these results indicate impaired citrate metabolism and transport in the lungs and MLE12 cells stimulated by LPS.

### Rescuing citrate^mt^ metabolism and transport attenuates LPS-induced necroptosis in AECs

**Fig. 3:**
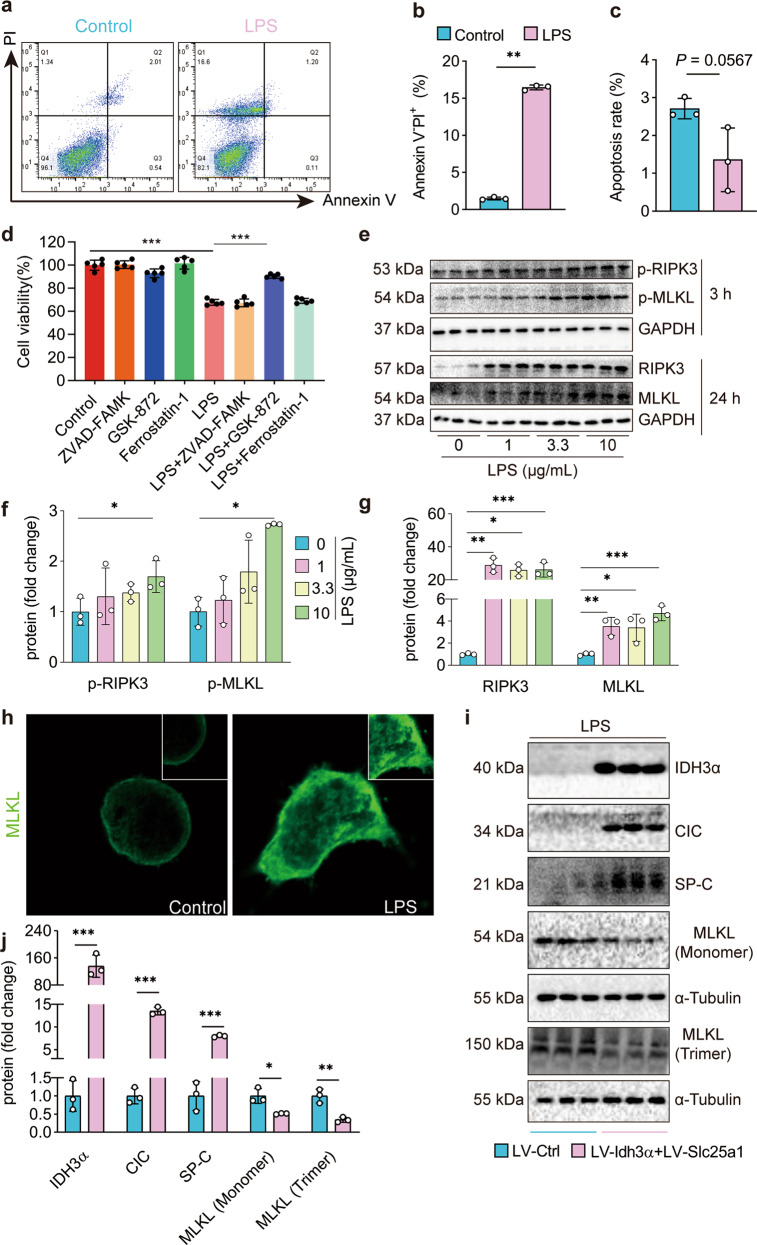
Rescued citrate^mt^ metabolism and transport attenuate LPS-induced necroptosis in AECs. **a**–**c** Apoptosis and necrosis in LPS-treated MLE12 cells were analyzed by flow cytometry (*n* = 3). **d** The RIPK3 inhibitor but not the apoptosis or ferroptosis inhibitor restored cell viability within 24 h of LPS incubation (*n* = 3). **e**–**g** The immunoblots show elevated expression and phosphorylation of the necroptosis-related proteins RIPK3 and MLKL (*n* = 3). **h** Immunofluorescence staining of MLKL (green) in LPS-treated MLE12 cells. **i**, **j** The immunoblots show that Idh3α and Slc25a1 overexpression in MLE12 cells rescued the expression of SP-C and greatly reduced necroptosis, as shown by the decreases in total and trimerized MLKL. The data are shown as the mean ± SD. *P < 0.05, **P < 0.01, and ***P < 0.001.

Programmed death in AECs, such as apoptosis, ferroptosis, and necroptosis, is crucial in the occurrence and development of ALI^38^. We found that LPS did not affect apoptosis- or ferroptosis-related genes or proteins, including BAX, BCL-2, Caspase-3 (markers of apoptosis), GPX4, and ACSL4 (markers of ferroptosis) (Supplementary Fig. 2a–d). Interestingly, LPS increased the percentage of necrotic/dead cells but not the percentage of apoptotic cells (Fig. 3a–c). In addition, the RIPK3 inhibitor but not the ferroptosis or apoptosis inhibitor rescued the LPS-induced reduction in MLE12 cell viability (Fig. 3d). An RT‒PCR array assay and gene ontology and pathway enrichment analysis revealed that genes involved in necroptosis are upregulated in LPS-induced lung injury^9^. Our results demonstrated that LPS significantly increased the protein expression and phosphorylation levels of RIPK3 and MLKL (Fig. 3e–g), and LPS induced trimerized MLKL translocation to the plasma membrane to form pores (Fig. 3h). Taken together, these results suggest that LPS (0, 1, 3.3, and 10 μg/mL) induced necroptosis but not apoptosis or ferroptosis in AECs.

To investigate whether citrate^mt^ accumulation had any effect on LPS-induced necroptosis, we generated MLE12 cells that overexpressed Idh3α and Slc25a1. We found that the overexpression of Idh3α and Slc25a1 rescued the protein expression of surfactant protein C (SP-C) and significantly reduced necroptosis, as shown by the decreased protein expression of RIPK3 and MLKL and levels of trimerized MLKL in MLE12 cells treated with LPS (Fig. 3i, j). Collectively, these results suggest that restoring citrate^mt^ metabolism and transport attenuates LPS-induced necroptosis in AECs.

### Citrate^mt^ accumulation drives necroptosis but not apoptosis or ferroptosis in AECs

**Fig. 4:**
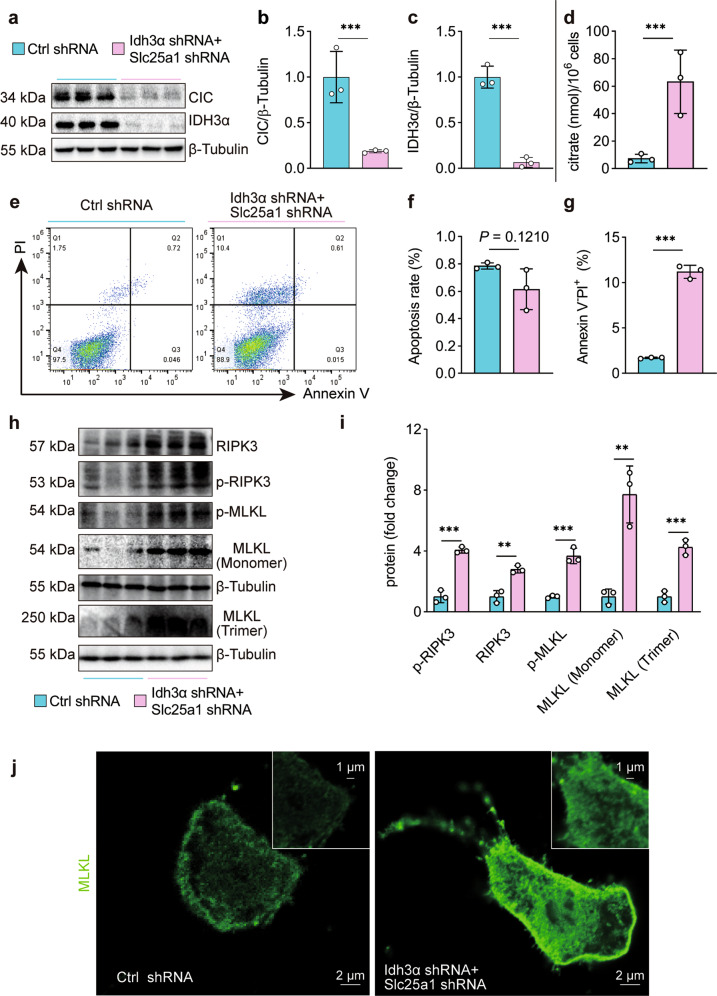
Necroptosis but not apoptosis or ferroptosis is promoted by citrate^mt^ accumulation induced by Slc25a1 and Idh3α shRNA in vitro. **a**–**c** The citrate^mt^ accumulation model was established by silencing Slc25a1 and Idh3α with shRNA in MLE12 cells (*n* = 3). **d** Citrate was measured with a citrate assay kit (*n* = 3). **e**–**g** Apoptotic and necrotic cells were assessed by flow cytometry with annexin V and PI staining (*n* = 3). **h**, **i** The immunoblots showed elevated expression of the necroptosis-related proteins RIPK3 and the MLKL monomer and increased levels of phosphorylated and trimerized MLKL (*n* = 3). **j** Immunofluorescence staining of MLKL (green) in MLE12 cells with citrate^mt^ accumulation. The data are shown as the mean ± SD. *P < 0.05, **P < 0.01, and ***P < 0.001.

To further determine the effects of citrate^mt^ accumulation on the death of AECs, we established a citrate^mt^ accumulation model with an Idh3α-silencing lentivirus (Idh3α shRNA) and a Slc25a1-silencing lentivirus (Slc25a1 shRNA) (Supplementary Fig. 3a–d). Simultaneous infection with Idh3α shRNA and Slc25a1 shRNA induced citrate^mt^ accumulation in MLE12 cells (Fig. 4a–d) and downregulated SP-C protein expression (Supplementary Fig. 3e). Importantly, immunoblotting and flow cytometry demonstrated that citrate^mt^ accumulation did not affect AEC apoptosis, whereas it increased the percentage of necrotic cells (Fig. 4e–g). The abundance of ferroptosis-related proteins, such as GPX4 and ACSL4, did not change with citrate^mt^ accumulation (Supplementary Fig. 3f).

To understand the biological role of citrate^mt^ accumulation, we performed RNA-seq analysis. We found that 215 genes were upregulated in MLE12 cells with citrate^mt^ accumulation (Supplementary Fig. 4a). To determine the functional properties of the top 30 genes that were upregulated by citrate^mt^, we performed Kyoto Encyclopedia of Genes and Genomes (KEGG) pathway analysis. KEGG pathway analysis indicated that the TNF signaling pathway was markedly increased in MLE12 cells with citrate^mt^ accumulation (Supplementary Fig. 4b), and the TNF signaling pathway is one of the critical signaling pathways that triggers necroptosis. For further validation, western blotting was performed. Interestingly, citrate^mt^ accumulation enhanced the expression of necroptosis-related proteins, including RIPK3 and MLKL monomers, and increased the levels of phosphorylated and trimerized MLKL (Fig. 4h, i). Citrate^mt^ accumulation also induced the translocation of trimerized MLKL to the plasma membrane to form pores, causing MLE12 cells to swell (Fig. 4j). We also established a citrate^mt^ accumulation model by chemically inhibiting IDH3 and CIC (Supplementary Fig. 5a, b). In this model, citrate^mt^ accumulation downregulated SP-C protein expression (Supplementary Fig. 5c) and significantly upregulated the expression and increased the phosphorylation of necroptosis proteins such as RIPK3 and MLKL (Supplementary Fig. 5d–g). Citrate^mt^ accumulation did not affect the levels of apoptosis or ferroptosis proteins (Supplementary Fig. 5f, g). Taken together, these results further suggest that citrate^mt^ accumulation specifically drives necroptosis.

### AEC-specific citrate^mt^ accumulation triggers necroptosis and exacerbates lung injury in mice

**Fig. 5:**
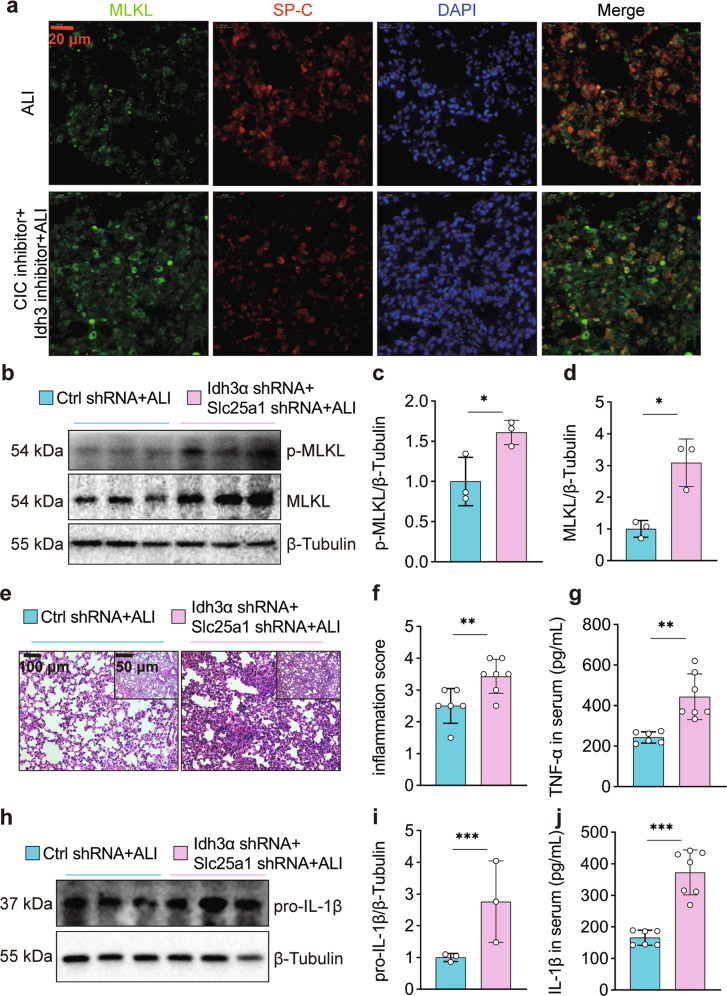
Citrate^mt^ accumulation amplifies necroptosis and exacerbates LPS-induced lung tissue injury. The mice were simultaneously injected with inhibitors of CIC and IDH3 or with sterile saline three days prior to intratracheal instillation of LPS (2.5 mg/kg) and were sacrificed 12 h after LPS administration. **a** Immunofluorescence staining and confocal microscopy were used to determine the localization of MLKL and SP-C. **b**–**d** Adenovirus vectors to specifically silence Slc25a1 and Idh3α in AECs were injected 10 days prior to intratracheal instillation of LPS (2.5 mg/kg), and the mice were sacrificed 12 h after LPS administration. MLKL and p-MLKL were detected by immunoblotting (*n* = 3). **e** Left lung tissue was embedded with paraffin and stained with hematoxylin and eosin (×100 and ×200 magnification). **f** The inflammation score was measured independently by three pathologists who were blinded to the experiment (*n* = 6–7). **g** Serum TNF-α levels were measured by ELISA (*n* = 6–7). **h**, **i** The expression of pro-IL-1β in the lungs was assessed by immunoblotting (*n* = 3). **j** Serum IL-1β levels were measured by ELISA (*n* = 6–7). The data are shown as the mean ± SD. *P < 0.05, **P < 0.01, and ***P < 0.001.

To investigate the effects of citrate^mt^ accumulation in AECs on lung tissue injury, we pretreated mice with IDH3 and CIC inhibitors (BTA and TBT) before intratracheal LPS administration. Compared with mice with LPS-induced ALI, mice that received LPS plus BTA and TBT had increased citrate^mt^ levels (Supplementary Fig. 6a), increased inflammation scores (Supplementary Fig. 6b, c), and higher left lung/weight ratios (Supplementary Fig. 6d). We also found that the expression and secretion of TNF-α and pro-IL-1β were increased in the serum and lungs (Supplementary Fig. 6e–h). The expression of SP-C was reduced, and the expression and phosphorylation of MLKL were elevated (Supplementary Fig. 6i). Immunofluorescence staining showed the colocalization of MLKL (green) and SP-C (red) in the lungs of ALI mice. MLKL fluorescence was increased, whereas SP-C fluorescence was decreased in the ALI + BAT + TBT group compared with the ALI group (Fig. 5a).

We then examined whether citrate^mt^ accumulation-induced lung injury depended on AEC-specific citrate^mt^ accumulation. To clarify this issue, we simultaneously injected SP-C promoter-adenovirus silencing vectors for Idh3α and Slc25a1 10 days prior to intratracheal instillation of LPS (2.5 mg/kg). We found that AEC-specific silencing of Idh3α and Slc25a1 further increased the expression and phosphorylation of MLKL (Fig. 5b–d) and further exacerbated the LPS-induced inflammatory response, including thickening of the alveolar walls, interstitial infiltration of inflammatory cells, and collapse of alveoli (Fig. 5e, f). We also found that the expression and secretion of TNF-α and pro-IL-1β were increased in the serum and lungs of mice at 12 h after LPS treatment (Fig. 5g–j). These results suggest that AEC-specific citrate^mt^ accumulation amplifies necroptosis and exacerbates lung tissue injury in mice.

### Citrate^mt^ accumulation leads to excessive mitophagy in AECs *via* DRP1

**Fig. 6:**
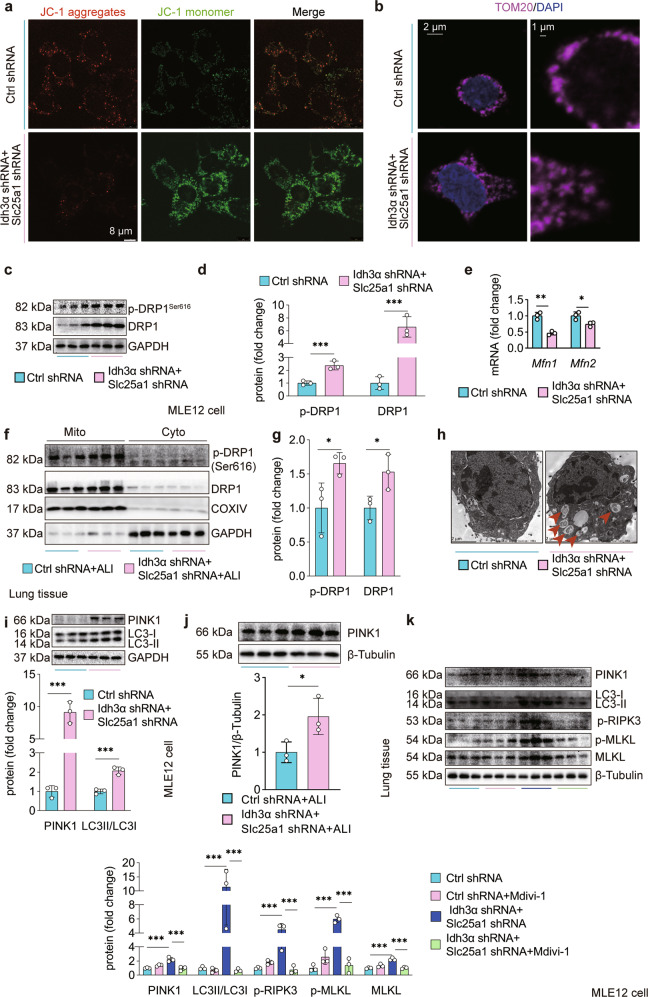
Citrate^mt^ accumulation induces excessive mitophagy *via* DRP1. **a** MMP was assessed by confocal laser scanning microscopy using the dye JC-1. **b** Confocal immunofluorescence staining of TOM20 was performed to visualize mitochondrial fission induced by citrate^mt^ accumulation. **c** *Mfn1* and *Mfn2* mRNA levels were measured by qPCR. **d**–**e** The total and Ser^616^ phosphorylated forms of DRP1 were measured by immunoblotting. **f**, **g** Adenovirus vectors (1 × 10^8^ PFU/20 g) to specifically silence Slc25a1 and Idh3α in AECs were injected 10 days prior to tracheal instillation of LPS (2.5 mg/kg), and the mice were sacrificed 12 h after LPS administration. Mitochondrial and cytosolic fractions were prepared. Silencing slc25a1 and Idh3α in AECs increased total and DRP1 protein in mitochondria (*n* = 3). **h** Autophagosome formation in mitochondria was detected by TEM. **i**, **j** The protein expression of PINK1 and LC3II/LC3I was measured by immunoblotting (*n* = 3). To observe the effects of DRP1 on mitophagy and necroptosis, MLE12 cells were treated with the DRP1-specific inhibitor Mdivi-1 (10 μM) for 1 h before lentiviral vector restimulation. **k** Mitophagy-related proteins (PINK1 and LC3II/LC3I) and necroptosis-related proteins (p-RIPK3, MLKL, and p-MLKL) were detected by immunoblotting (*n* = 3). The data are shown as the mean ± SD. *P < 0.05, **P < 0.01, and ***P < 0.001.

To determine the potential mechanisms underlying citrate^mt^ accumulation-induced necroptosis in AECs, we examined whether citrate^mt^ accumulation had any effect on mitochondrial function. We measured the MMP and observed that citrate^mt^ accumulation induced mitochondrial depolarization, as indicated by a decrease in the JC-1 red/green fluorescence intensity ratio (Fig. 6a). Confocal image analysis showed TOM20 staining (Fig. 6b), which indicated that citrate^mt^ accumulation initiated mitochondrial fission in vitro. Phosphorylation of DRP1 at Ser^616^ promotes DRP1 recruitment to mitochondria and subsequent fission^39^. We found increased expression of DRP1 and increased levels of p-DRP1^Ser616^ in MLE12 cells with citrate^mt^ accumulation (Fig. 6c, d), whereas mitochondrial fusion-related genes such as *Mfn1* and *Mfn2* were downregulated (Fig. 6e). Interestingly, we extracted mitochondrial and cytosolic proteins from lung tissue and found that AEC-specific citrate^mt^ accumulation increased the protein and phosphorylation level of mitochondrial DRP1 in vivo (Fig. 6f, g), which suggested that the increase in DRP1 in the lungs of ALI mice was associated with mitochondria.

DRP1-mediated mitochondrial fission contributes to excessive mitophagy, which leads to cell death^29,40^. DRP1 recruitment to mitochondria has a crucial function in PINK1/Parkin-mediated mitophagy activation^41^. Importantly, our TEM data showed that citrate^mt^ accumulation caused excessive autophagosome formation in mitochondria (Fig. 6h). Citrate^mt^ accumulation also upregulated the expression of the mitophagy-related protein PINK1 and increased the ratio of LC3-II/I, which is an autophagosome marker (Fig. 6i). Furthermore, AEC-specific citrate^mt^ accumulation upregulated the expression of PINK1 in the lungs of ALI mice (Fig. 6j). We found the same results in IDH3 + CIC inhibitor-treated MLE12 cells (Supplementary Fig. 7a, b). To evaluate the role of DRP1 in citrate^mt^ accumulation-induced mitophagy, we used Mdivi-1, a selective inhibitor of DRP1. We found that treatment with Mdivi-1 significantly reduced mitophagy and necroptosis in MLE12 cells following shRNA-mediated silencing of Idh3a and Slc25a1 (Fig. 6k). Collectively, these results indicate that the mitochondrial toxicity caused by citrate^mt^ accumulation in MLE12 cells and lung tissue is mainly induced by DRP1-mediated excessive mitophagy.

### Citrate^mt^ accumulation upregulates and recruits cytoplasmic DRP1 to mitochondria by directly binding to FUNDC1

**Fig. 7:**
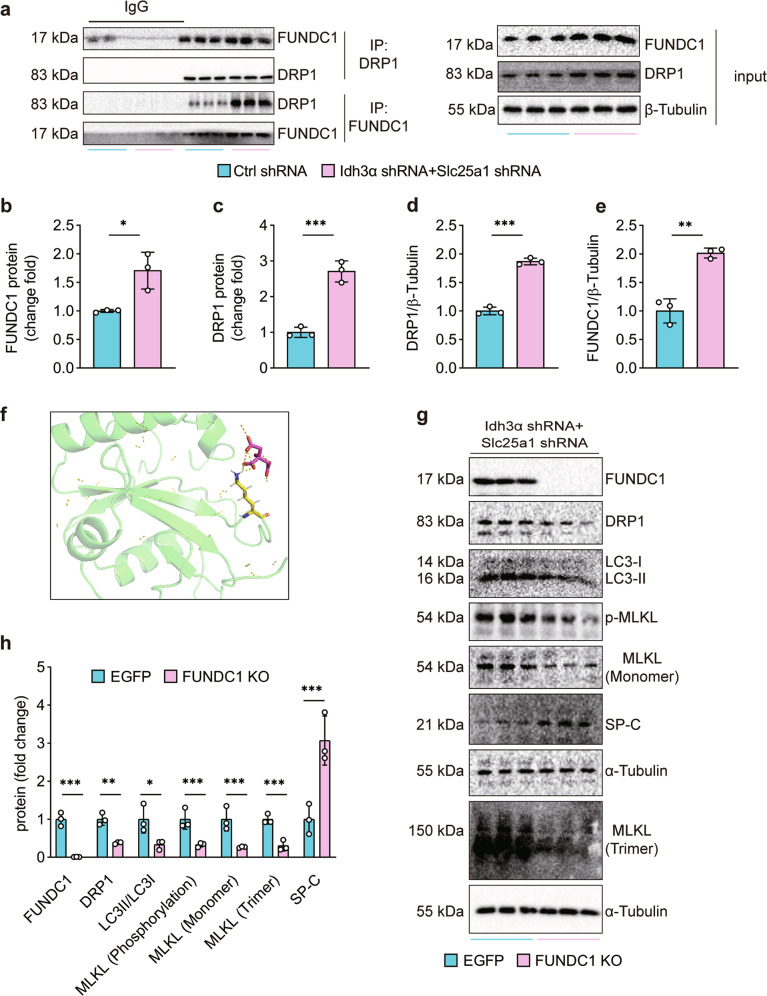
Citrate^mt^ accumulation recruits cytoplasmic DRP1 to mitochondria by direct binding to FUNDC1. **a**–**d** The interaction between FUNDC1 and DRP1 was detected by coimmunoprecipitation (*n* = 3). **a**, **e** Citrate^mt^ accumulation upregulated FUNDC1 protein expression (*n* = 3). **f** Molecular docking model of FUNDC1-citrate created by AutoDock and PyMOL. FUNDC1-KO MLE12 cells were generated *via* the CRISPR/Cas9 system. **g**, **h** Citrate^mt^ accumulation failed to increase the expression of DRP1 or increase the levels of LC3II/LC3I or trimerized MLKL in FUNDC1-KO MLE12 cells; SP-C protein expression was rescued in FUNDC1-KO MLE12 cells (*n* = 3). The data are shown as the mean ± SD. *P < 0.05, **P < 0.01, and ***P < 0.001.

We next examined how citrate^mt^ accumulation was associated with DRP1. Considering that FUNDC1 can recruit and activate DRP1^25^, we performed coimmunoprecipitation assays with anti-FUNDC1 and anti-DRP1 antibodies. As shown in Fig. 7a–d, citrate^mt^ accumulation strongly promoted the interaction between FUNDC1 and DRP1 and upregulated FUNDC1 levels in MLE12 cells (Fig. 7a, e). The protein expression of FUNDC1 in lungs and MLE12 cells was also upregulated in response to LPS challenge (Supplementary Fig. 8a, b). To further determine whether citrate could bind with FUNDC1, we used AutoDock and PyMOL software to predict and visualize the interaction of citrate with FUNDC1 (Fig. 7f). The results showed that citrate^mt^ could directly interact with FUNDC1. To confirm that FUNDC1 was required for citrate^mt^ accumulation-induced necroptosis, we generated a FUNDC1-KO MLE12 cell line with CRISPR/Cas9. Furthermore, we observed that the protein expression of DRP1 and trimerized MLKL and the levels of LC3II/LC3I were greatly reduced, whereas SP-C expression was increased in FUNDC1-KO MLE12 cells in response to citrate^mt^ accumulation (Fig. 7g, h). Collectively, these data demonstrate that citrate^mt^ directly binds to FUNDC1, which exacerbates DRP1-mediated mitochondrial fission.

## Discussion

The present study used gain- and loss-of-function approaches to reveal that citrate^mt^ accumulation significantly contributed to AEC necroptosis during ALI, while a reduction in citrate^mt^ protected AECs against LPS-induced necroptosis. Mechanistically, we identified that accumulated citrate^mt^ interacted with DRP1 through FUNDC1 and enhanced mitochondrial fission, resulting in excessive mitophagy and triggering necroptosis in AECs, ultimately initiating and promoting the development of ALI/ARDS. We believe that our study uncovered a novel functional role for citrate^mt^ accumulation as a critical modulator of the FUNDC1/DRP1/excessive mitophagy axis in the lung, which may provide new strategies for treating AEC injury after exposure to LPS during ALI/ARDS.

Glucose metabolism is an essential energy and material source for cells and maintaining glucose homeostasis is essential in response to the spatiotemporal variability of environmental factors. Previously, we showed that glucose metabolic reprogramming occurred in the lungs in LPS-induced ALI^37^. To identify the mechanism, we measured glucose metabolic products. Pyruvate and citrate, which are key substrates of the TCA cycle, both accumulated. To verify the in vivo results, we observed that citrate accumulated in MLE12 cells after LPS challenge, suggesting that citrate may be a damaging factor that triggers ALI.

An in-depth investigation of the molecular mechanisms underlying citrate^mt^ accumulation-induced injury is required to understand LPS-induced ALI pathogenesis. Citrate is produced by the TCA cycle, where it is decarboxylated to generate α-KG by IDH3 or transported into the cytoplasm by CIC. Thus, the expression of IDH3 and CIC is highly correlated with citrate^mt^ homeostasis. Decreased expression of IDH3 and CIC suggests citrate^mt^ accumulation, which was confirmed by measuring citrate^mt^ levels. Citrate^mt^ is both an intermediate of the TCA cycle and a signaling molecule. For example, citrate^mt^ directly inhibits the activities of succinate dehydrogenase and pyruvate dehydrogenase^42^. Here, we found that citrate^mt^ induced AEC injury.

Kitamura et al. showed that Fas/Fas ligand-dependent AEC apoptosis occurred in mouse models of ARDS^43^. Necrosis regulation is involved in the pathogenesis of experimental ARDS^44^. Consistent with these reports, we found that 1, 3.3, and 10 μg/mL LPS induced necroptosis but not apoptosis or ferroptosis in MLE12 cells. Necroptosis is a caspase-independent form of lytic programmed cell death. Aberrant activation of necroptosis is implicated in many pathologies. The necroptosis pathway relies on RIPK3 activation^45^, which causes the phosphorylation and oligomerization of MLKL, which translocate to the plasma membrane, where it forms membrane-disrupting pores and leads to cell lysis^46^. We found that citrate^mt^ accumulation increased necroptosis-related proteins in AECs. In this work, we did not examine the effects of citrate^mt^ accumulation on pyroptosis because pyroptosis mostly occurs in macrophages, endothelial cells, and neutrophils^47^ during ALI. Collectively, these data indicate that citrate^mt^ induces AEC injury *via* necroptosis.

Mitochondria are highly dynamic, double-membrane organelles that participate in many functions within eukaryotic cells^48^. Maintaining mitochondrial health is essential for the survival and functions of eukaryotic organisms^49^. Mitochondrial homeostasis is ensured by coordinating mitochondrial biogenesis, mitochondrial fission, mitochondrial fusion, and mitophagy^50^. Mitochondrial fission, which is mediated by the cytosolic dynamin family member DRP1, is essential for growing and dividing cells to populate them with adequate numbers of mitochondria. DRP1 is recruited from the cytosol to form spirals around mitochondria that constrict and sever both the inner and outer membranes and then trigger fission^51^. However, excessive mitochondrial fission is an early marker of mitochondrial damage^29^, which leads to persistent mitochondrial loss, energy shortages, oxidative stress, and ultimately cell death^52^. DRP1-dependent mitochondrial fission is a prerequisite for mitophagy^52^. Mitophagy is an important protective mechanism for the selective removal of mitochondria to maintain cellular homeostasis in response to pathological conditions^53^. However, excessive mitophagy is maladaptive and linked to cell death^28,40^, such as cigarette smoke exposure-induced mitophagy-dependent necroptosis in lung emphysema^39^. DRP1-mediated mitochondrial fission induces LC3B lipidation and mitophagy, which requires Parkin/PTEN-induced kinase 1 (PINK1)^28^. AECs with accumulated citrate^mt^ exhibited excessive autophagosome formation, and citrate^mt^ upregulated the expression of PINK1 and increased the ratio of LC3-II/LC3-I, suggesting that elevated citrate^mt^ induced excessive AEC mitophagy *via* DRP1 and triggered necroptosis.

The mitochondrial-associated membrane protein FUNDC1 recruits DRP1 to drive mitochondrial fission in response to hypoxic stress^54^. Overexpression of FUNDC1 promotes mitochondrial dysfunction in cardiomyocytes exposed to normal glucose^55^, whereas silencing FUNDC1 alleviates chronic obstructive pulmonary disease by inhibiting mitophagy^56^. Here, we showed that the accumulation of citrate^mt^ promoted the interaction between DRP1 and FUNDC1. Citrate can inhibit PNPase by binding to the active sites of two distantly related PNPases^34^. The AutoDock and PyMOL results demonstrated that citrate could directly bind to FUNDC1, suggesting that accumulated citrate^mt^ recruits cytoplasmic DRP1 to mitochondria by directly binding to FUNDC1, consequently initiating the recruitment of DRP1 to drive mitochondrial fission, which leads to excessive mitophagy and ultimately triggers necroptosis in AECs during ALI.

Although citrate can inhibit PNPase by binding to the active sites of two distantly related PNPases^34^, in our study, the binding of FUNDC1 to citrate was predicted by PyMOL, and the binding of FUNDC1 to citrate should be more carefully confirmed using purified proteins in the future. In addition, this study focused on the molecular mechanism of citrate^mt^ accumulation-induced AEC death. Unlike macrophages, AECs are challenging to obtain from ARDS patients, especially healthy volunteers. Therefore, we have no ARDS patient data to confirm the role of citrate^mt^ accumulation in AECs death. Single-cell RNA sequencing may provide more accurate clues. Finally, even though TBT and BTA could suppress IDH3 or CIC, they lack specificity. Therefore, specific small molecules should be further screened. The inhalation of CIC and IDH3 agonists may be an effective strategy for attenuating the loss of AECs during ARDS.

**Fig. 8:**
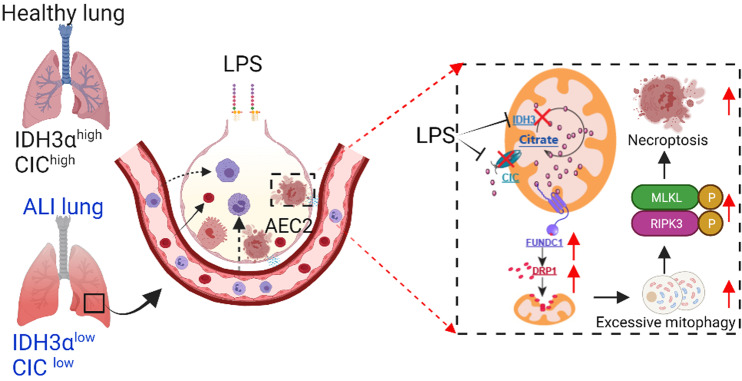
Schematic diagram showing citrate^mt^ accumulation-mediated necroptosis in AECs. LPS downregulates Idh3α and Slc25a1, leading to citrate^mt^ accumulation in AECs. Citrate^mt^ accumulation induces DRP1-mediated mitochondrial fission and excessive mitophagy *via* FUNDC1, which damages mitochondria in AECs. As a consequence of RIPK3 activation, MLKL is phosphorylated and activated, which leads to AEC necroptosis and ALI/ARDS.

In conclusion, our data demonstrate that accumulated citrate^mt^ directly binds to FUNDC1, recruits cytoplasmic DRP1 to mitochondria and exacerbates mitochondrial fission, resulting in excessive mitophagy and triggering necroptosis in AECs, ultimately initiating and promoting the development of ALI/ARDS (Fig. 8). We provide evidence that citrate^mt^ accumulation is associated with LPS-induced ALI. We suggest that targeting citrate^mt^ accumulation may be an additional therapeutic approach to reduce ALI/ARDS morbidity.

## Supplementary information

Supplementary

## Data Availability

The data supporting the results of this study are available within the Source data files provided with this paper. The untargeted metabolomics data are available through with the following accession number: MTBLS4379. The RNA-seq data have been deposited in GEO with the following accession number: GSE197793.
