# Supplementary material for: Mitochondrial citrate accumulation drives alveolar epithelial cell necroptosis in lipopolysaccharide-induced acute lung injury
Source: Exp Mol Med. 2022 Nov 28;54(11):2077–91. doi: 10.1038/s12276-022-00889-8 (PMC9722936; doi:10.1038/s12276-022-00889-8)
Supplement: Supplementary file 1 — Supplementary [file 12276_2022_889_MOESM1_ESM.pdf]

Materials and Methods

RNA-Seq Gene Expression Analysis

The transcriptome sequencing experiments were performed by Genechem Co., LTD. (Shanghai, China). Briefly, total RNA was extracted from control and Slc25a1 plus Idh3α-deficient MLE12 cells. The transcriptome library for sequencing was generated using NEBNext® Ultra™ RNA Library Prep Kit for Illumina®, following the manufacturer's recommendations. The index codes were added to attribute sequences to each sample. After the quality of sequencing libraries was confirmed on the Agilent 2100 system, clustering of the index-coded samples was performed on a cBot Cluster Generation System using TruSeq PE Cluster Kit v3-cBot-HS (Illumia, San Diego, CA, USA) according to the manufacturer's instructions. The library preparations were then sequenced on an Illumina Hiseq 2500 platform (Illumina, San Diego, CA, USA), and paired-end reads were generated. Transcriptome assembly was accomplished based on the left.fq and right.fq using Trinity with min_kmer_cov set to 2 by default and all other parameters set to default.

Supplementary Tables

Supplementary Table1. Sequences of the primers used to quantitate gene expression

Gene	Forward primer (5′–3′)	Reverse primer (5′–3′)
Idh3α	TTGCTGGTGGTGTTCAGACA	ATTGCTGTGACATTGCGCTC
Sdhα	AGAGATACGCACCTGTTGCC	ACTGGGATGGGCTCCTTAGT
Slc25a1	TGCAGCCAGTGTCTTTGGAA	AGGATCTTCAAGCCGCAGTC
Acly	TTCCTCCTTAATGCCAGCGG	AGGGATCTTGGACTTGGGACT
Mfn1	CCTACTGCTCCTTCTAACCCA	AGGGACGCCAATCCTGTGA
Mfn2	CGGTTCACTGTACCCCACTT	GAGGCCAGTAGTGTTGCCTT
Sftpb	TGCCCCTGGTTATTGACTACTT	CCTGGATTCTGTTCTGGCTTAG
Bax	GTCCACGTCAGCAATCATCC	GAGACACCTGAGCTGACCTT
Bcl-2	AAACCCTCCATCCTGTCCAG	CCCTTTCCTAGACCCAGCAA
Caspase-3	GAGCTTGGAACGGTACGCTAA	GAGTCCACTGACTTGCTCCC
β-actin	TTCCAGCCTTCCTTCTTG	GGAGCCAGAGCAGTAATC

Supplementary Table2. antibodies for Western blotting, Immunofluorescence, and IP assay

Antibodies	Source	Catalog	Dilution ratio
**Primary antibodies for Western blotting**			
**Rabbit anti-CIC polyclonal antibody**	Thermo-Fisher	PA5-85163	1: 2000
**Rabbit anti-IDH3 polyclonal antibody**	Abcam	Ab228596	1: 2000
**Rabbit anti-MLKL polyclonal antibody**	Abcam	Ab172868	1: 2000
**Rabbit anti-phospho-MLKL phospho-S345 monoclonal antibody**	Abcam	Ab196436	1: 2000
**Rabbit-anti-RIPK3 polyclonal antibody**	Abcam	Ab62344	1: 2000
**Rabbit anti-phospho-RIPK3 phospho-S232 monoclonal antibody**	Abcam	Ab195117	1: 2000
**Rabbit anti-Caspase-8 polyclonal antibody**	Affinity	Ab-AF6442	1: 2500
**Rabbit anti-DRP1 monoclonal antibody**	Abcam	Ab184247	1: 2000
**Rabbit anti-phospho-DRP1 phospho-S616**	Cell Signaling Technology	# 3455	1: 2500
**Rabbit anti-PINK1 polyclonal antibody**	Abcam	Ab23707	1: 2000
**Rabbit anti-LC3II/LC3I monoclonal antibody**	Cell Signaling Technology	# 12741	1: 2000
**Rabbit anti-FUNDC1 monoclonal antibody**	Cell Signaling Technology	#49240	1: 2000
**Rabbit anti-GPX4 polyclonal antibody**	ABclonal	A1933	1: 2000
**Rabbit anti-ACSL4 monoclonal antibody**	Abcam	Ab155282	1: 2000
**Rabbit anti-BCL-2 polyclonal antibody**	Cell Signaling Technology	#3498	1: 2000
**Rabbit anti-BAX polyclonal antibody**	Cell Signaling Technology	#2772	1: 2000
**Rabbit anti-GAPDH polyclonal antibody**	Servicebio	GB11002	1: 2000
**Rabbit anti-α-Tubulin polyclonal antibody**	Servicebio	GB11200	1: 7500
**Rabbit anti-β-Tubulin polyclonal antibody**	Servicebio	GB11017	1: 7500
**Secondary antibodies for Western blotting**			
**HRP-conjugated goat anti-rabbit IgG**	Signalway Antibody	#L3012-2	1: 5000
**Primary antibodies for Immunofluorescence**			
**Rabbit anti-TOM20 polyclonal antibody**	Proteintech	# 11802-1-AP	1: 200
**Mouse anti-MLKL monoclonal antibody**	Proteintech	66675-1-Ig	1: 200
**Rabbit anti-SP-C polyclonal antibody**	Affinity	DF6647	1: 200
**Secondary antibodies for Immunofluorescence**			

**FITC goat anti-mouse IgG (H+L)**	Abclonal	# AS001	1: 400
**Rhodamine (TRITC) goat anti-rabbit IgG (H+L)**	Abclonal	#AS040	1: 400
**Cy5-conjugated goat anti-mouse IgG (H+L)**	Servicebio	GB27301	1: 400
**Antibodies for IP assay**			
**Rabbit anti-DRP1 monoclonal antibody**	Cell Signaling Technology	#8570	1: 100
**Rabbit anti-FUNDC1 monoclonal antibody**	Cell Signaling Technology	#49240	1: 200

Supplementary Figures and Figure legends

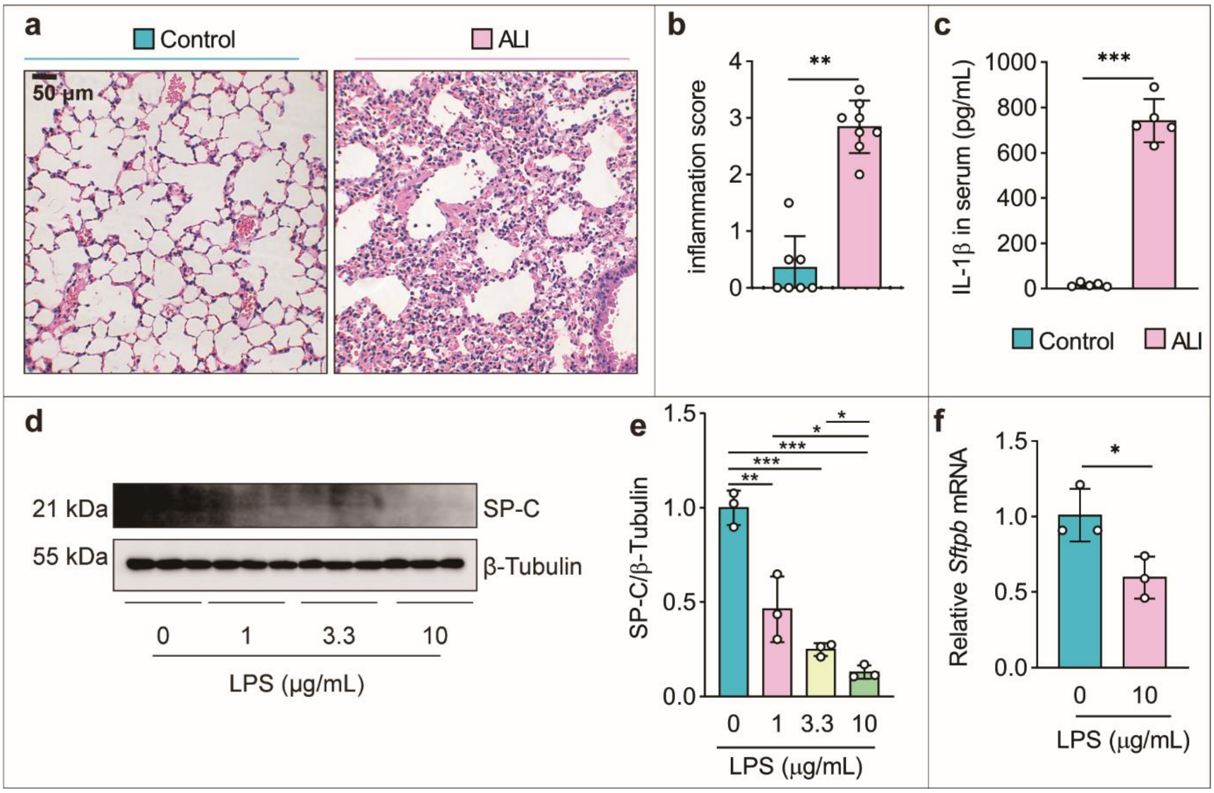

Supplementary Fig. 1. LPS induces ALI in mice and damages AEC. a. The ALI mice were injected intratracheally with LPS (5 mg/kg, *n*=8) or physiological saline (Control group, *n*=7). Histology (H&E staining) of the right upper lung sections (*n*=7-8, scale bars=50 μm). b. Inflammation score was used to quantify the degree of lung injury. Compared with controls, mice given LPS had increased inflammation scores (*n*=7-8). c. IL-1β content in serum was measured to quantify inflammation. Compared with controls, mice given LPS had increased secretion of IL-1β (*n*=7-8). d-f. The MLE12 cells were treated with an LPS concentration gradient from 0-10 μg/mL for 12 h (for gene detection) or 24 h (for protein detection). The expression of SP-C was measured to quantify AEC injury. Compared with controls, MLE12 cells given LPS had reduced protein expression of SP-C (*n*=3) and mRNA expression of *Sftp-b* (*n*=3). Data are shown as mean ± SD. * $P < 0.05$, ** $P < 0.01$, and *** $P < 0.001$.

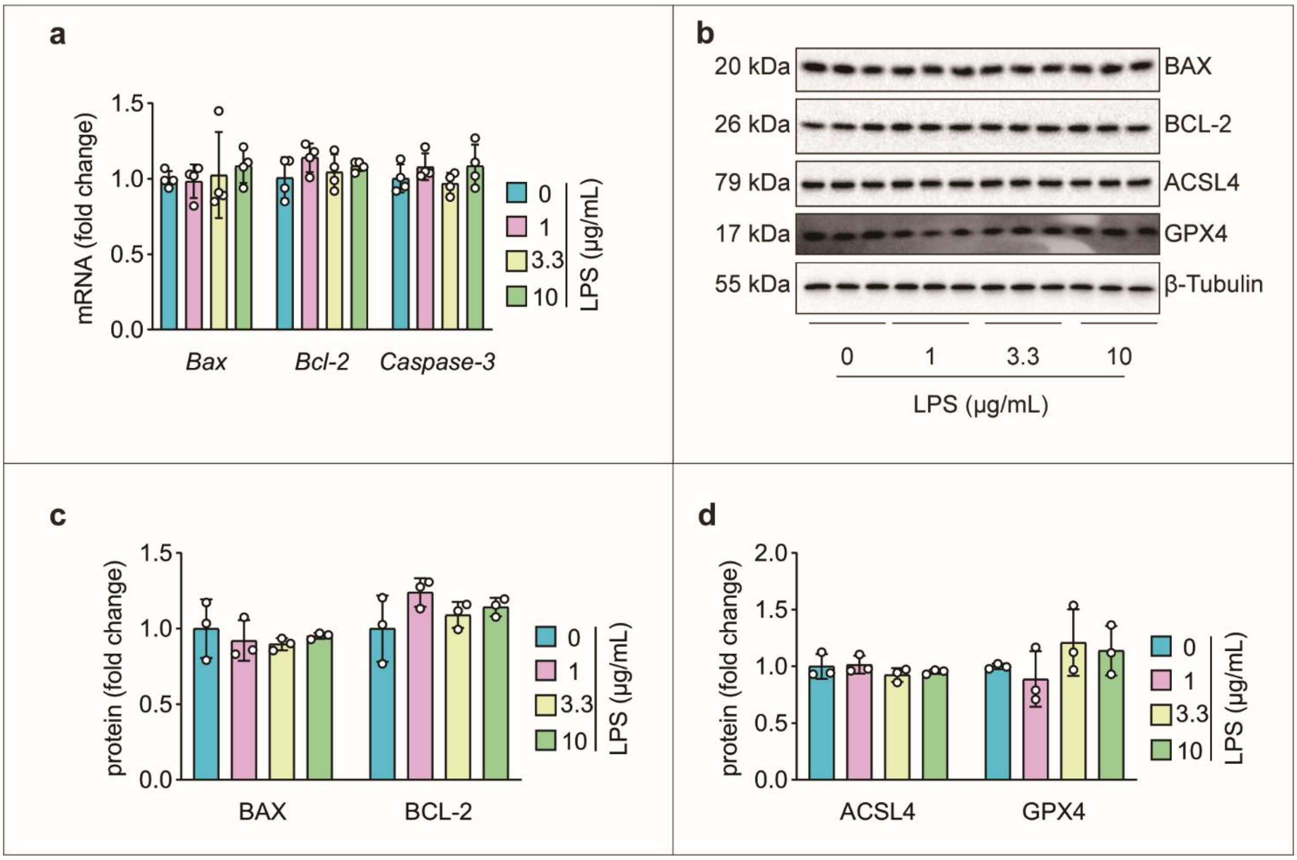

Supplementary Fig. 2. Indicated concentrations of LPS don't affect apoptosis or ferroptosis related-protein expression in MLE12 cells. The MLE12 cells were treated with an LPS concentration gradient from 0-10 μg/mL for 12 h (gene) or 24 h (protein). BAX, BCL-2, and Caspase-3 were measured to quantify the apoptosis. a. Compared with control, MLE12 cells given indicated concentration of LPS were unaffected in mRNA expression of *Bax*, *Bcl-2*, and *Caspase-3* (*n*=3). b-c. Protein expression was concordant with the qRT-PCR results. Compared with controls, MLE12 cells given indicated concentration of LPS were unaffected in protein expression of BAX or BCL-2 (*n*=3). d. Expression of ACSL4 and GPX4 was measured to quantify ferroptosis of AEC. Compared with the control, MLE12 cells given LPS were unaffected in protein expression of ACSL4 or GPX4 (*n*=3). Data are shown as mean ± SD.

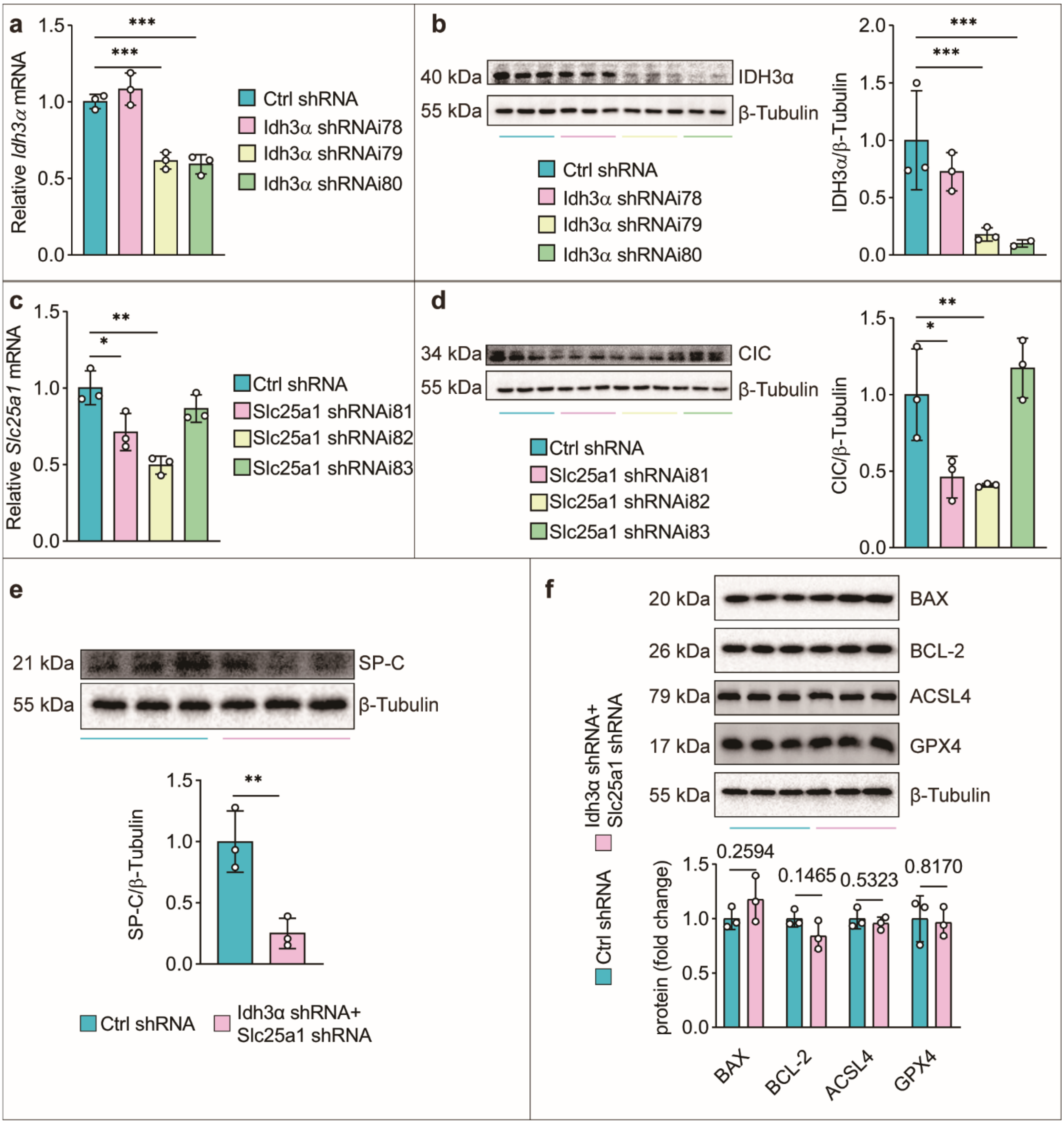

Supplementary Fig. 3. Effects of three pairs of shRNA expression vectors on Idh3α and CIC gene and protein expression. Three pairs of shRNA expression vectors were transfected into MLE12 cells for 16 h. The cells were washed and cultivated for an additional 80 h. a-b. Compared with the control group, Idh3α gene and protein expression were decreased in the shRNAi79 and shRNAi80 groups (*n*=2-3). c-d. CIC gene and protein expression were decreased in the shRNAi81 and shRNAi82 groups (*n*=3). e-f. Compared with controls, MLE12 cells given Idh3α shRNA+Slc25a1 shRNA had reduced protein expression of SP-C (*n*=3), whereas there was no effect on protein expression of BAX, BCL-2,

ACSL4, or GPX4 (n=3). Data are shown as the mean ± SD. * $P < 0.05$, ** $P < 0.01$, and *** $P < 0.001$.

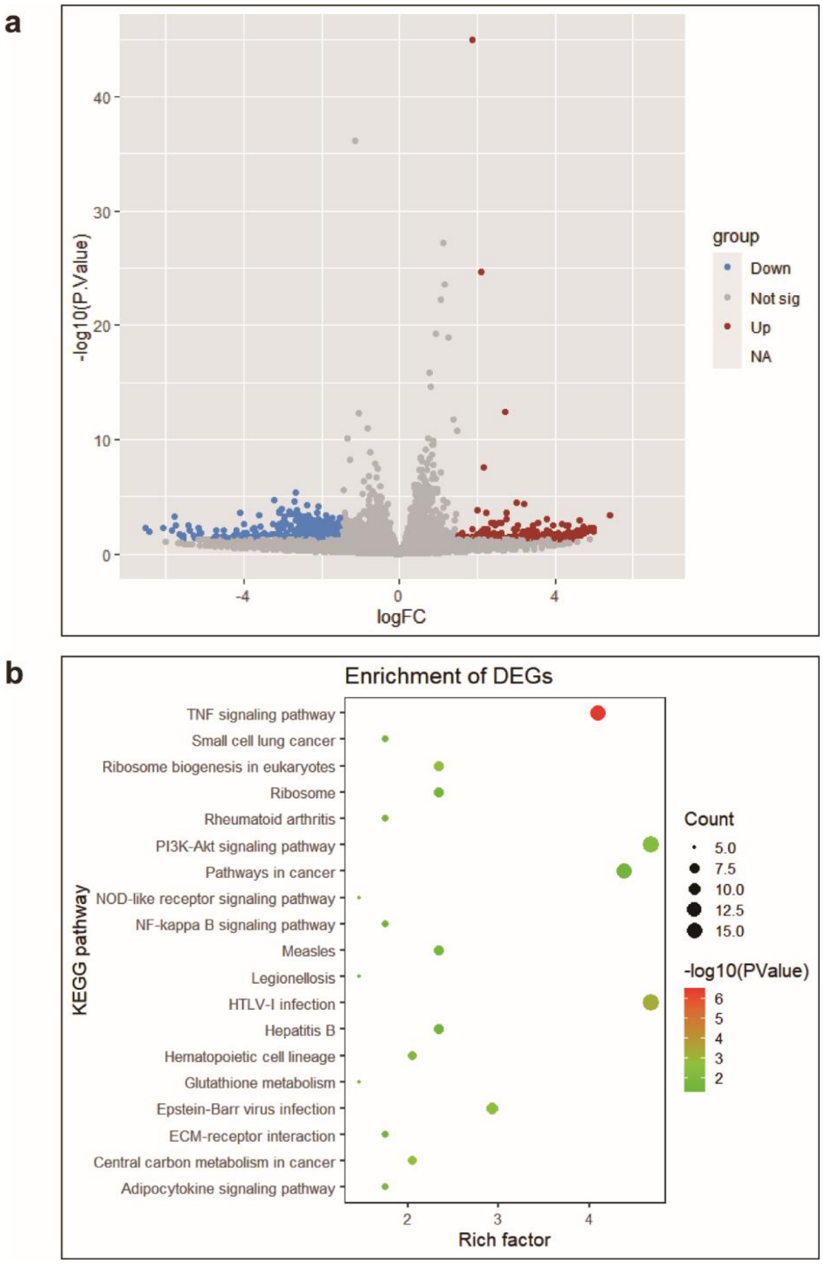

Supplementary Fig. 4. Transcription profiles of citrate^{mt}-accumulated cells. a. Volcano map depicting genes upregulated (red) or downregulated (blue) 2-fold or more in MLE12 cells on 8 dpi. b. Kyoto Encyclopedia of Genes and Genomes (KEGG) pathway in citrate^{mt}-accumulated MLE12 cells compared with control shRNA.

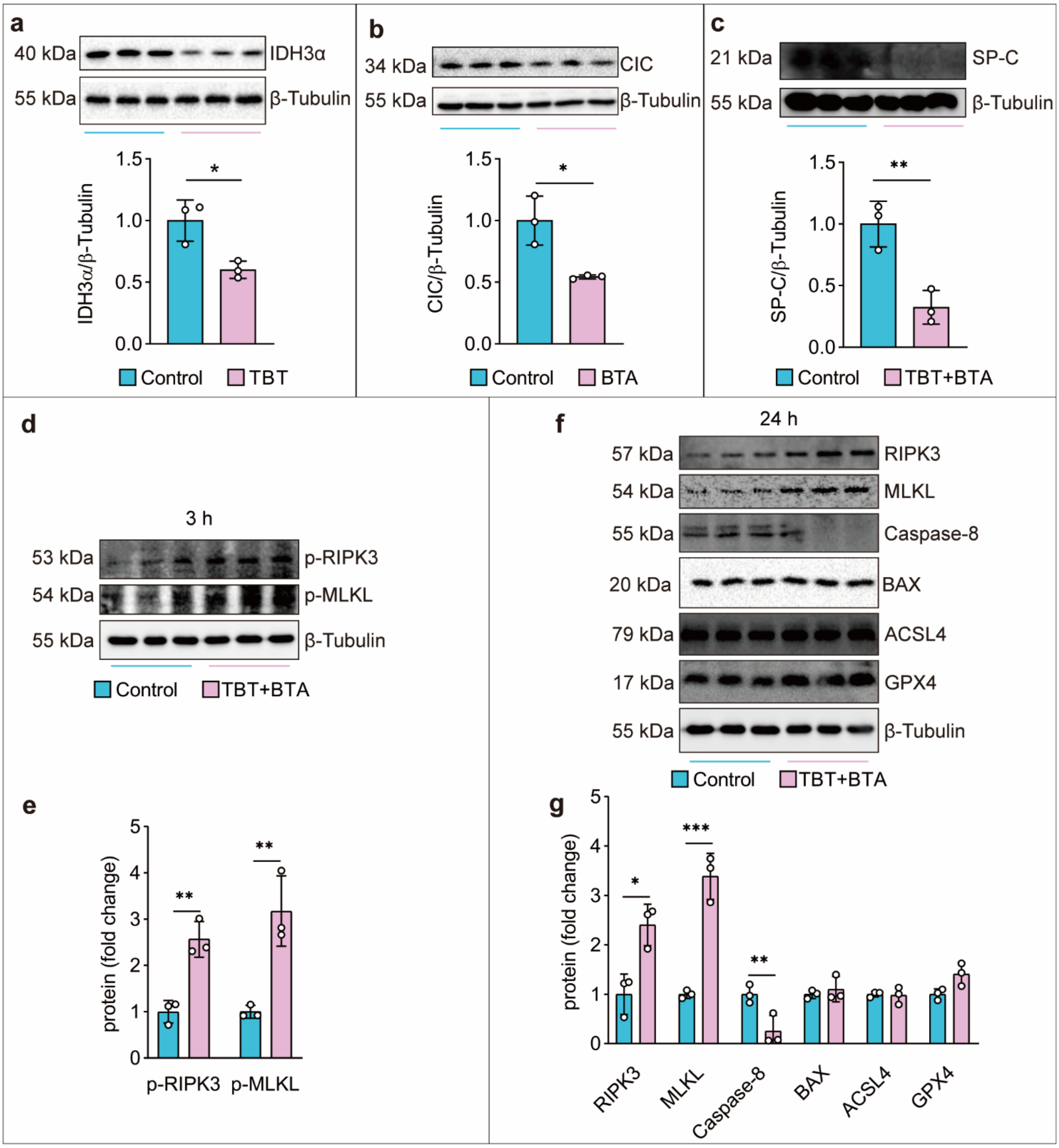

Supplementary Fig. 5. Simultaneous inhibition of IDH3 and CIC induces citrate^{mt} accumulation and damages AEC. a-b. MLE12 cells were treated simultaneously with the IDH3 inhibitor TBT (100 nM) and CIC inhibitor BTA (2 mM) or PBS for 24 h. The effects of inhibition were analyzed by immunoblotting (*n*=3). c. Expression of SP-C was measured to quantify AEC injury. Compared with controls, MLE12 cells simultaneously given TBT and BTA had reduced protein expression of SP-C (*n*=3). d-g. MLE12 cells simultaneously given TBT and BTA had increased amounts of p-RIPK3 and p-MLKL (*n*=3), augmented protein expression of RIPK3 and MLKL, and reduced expression of

Caspase-8, whereas there were no effects on BAX, ACSL4, or GPX4 (n=3). Data are shown as mean ± SD. * $P < 0.05$, ** $P < 0.01$, and *** $P < 0.001$.

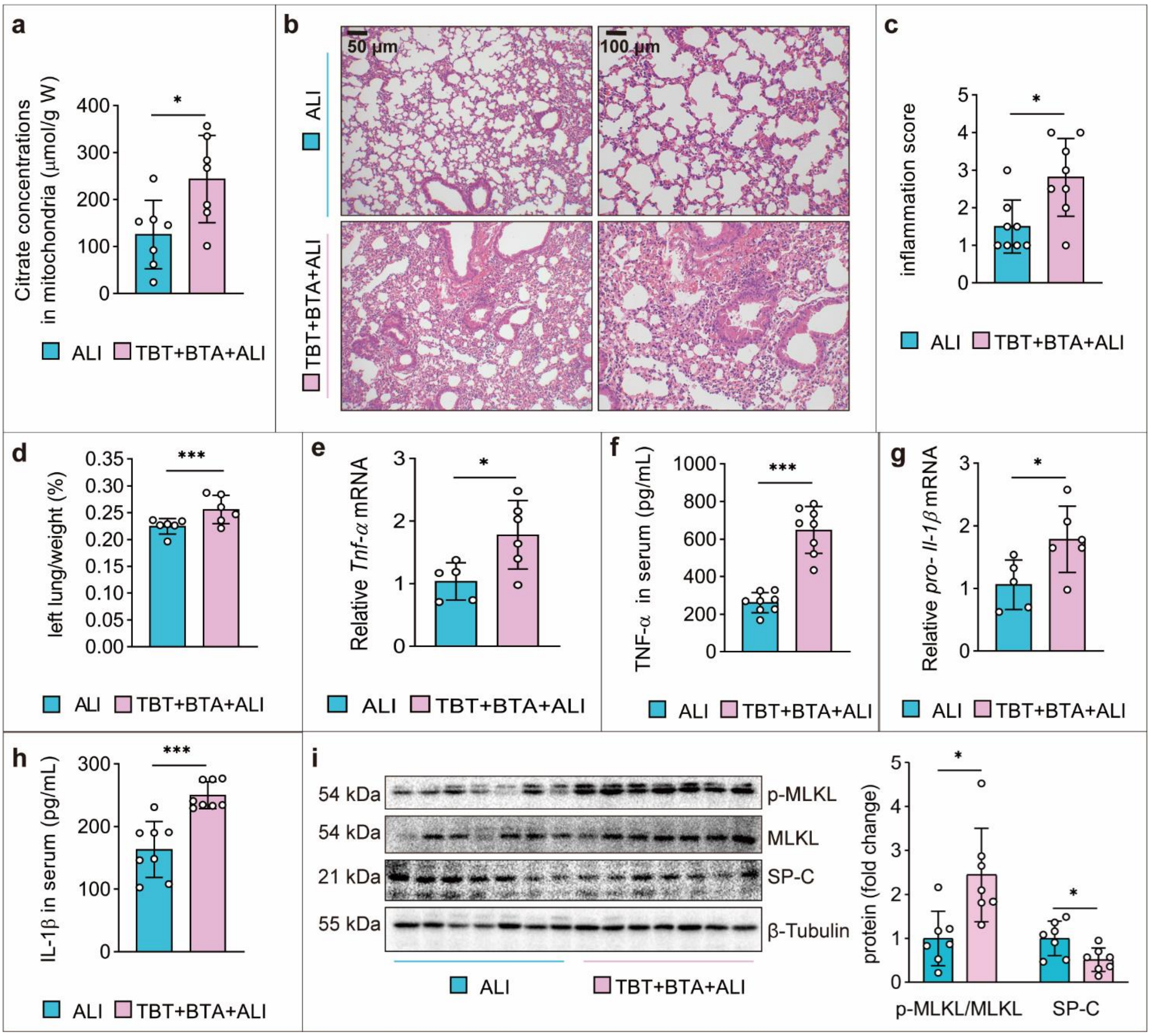

Supplementary Fig. 6. Inhibition of IDH3 and CIC expression aggravates lung tissue injury and amplifies inflammation and necroptosis. Mice were intraperitoneally injected with both IDH3 inhibitor (TBT, 20 mg/kg body weight) and CIC inhibitor (BTA 20 mg/kg body weight) or physiological saline every day for three days. Then mice were intratracheally injected with LPS (2.5 mg/kg, *n*=7) for 12 h. a. Compared with LPS-induced ALI, mice given LPS plus BTA and TBT had increased content of citrate^{mt} (*n*=7). b. Histology (H&E staining) of the right upper lung sections (*n*=8, Scale bars=50 or 100 μm). c. Inflammation score was used to quantify the degree of lung injury. Compared with ALI, mice receiving ALI+TBT+BTA had increased inflammation scores (*n*=8). d. Left lung/weight ratio was used to quantify the degree of pulmonary edema. Compared with ALI, mice

given ALI+TBT+BTA had an increased left lung/weight ratio (n=6). e-h. The expression and secretion of TNF-α and IL-1β in the lungs and serum were measured to quantify inflammation. Compared with ALI, mice given ALI+TBT+BTA had increased expression and secretion of TNF-α and IL-1β (n=5-8). Phospho-MLKL and MLKL were used to quantify necroptosis, and SP-C was measured to quantify AEC injury. i. Compared with ALI, mice given ALI+TBT+BTA had increased p-MLKL/MLKL ratio and reduced expression of SP-C (n=7). Data are shown as mean ± SD. * $P < 0.05$, ** $P < 0.01$, and *** $P < 0.001$.

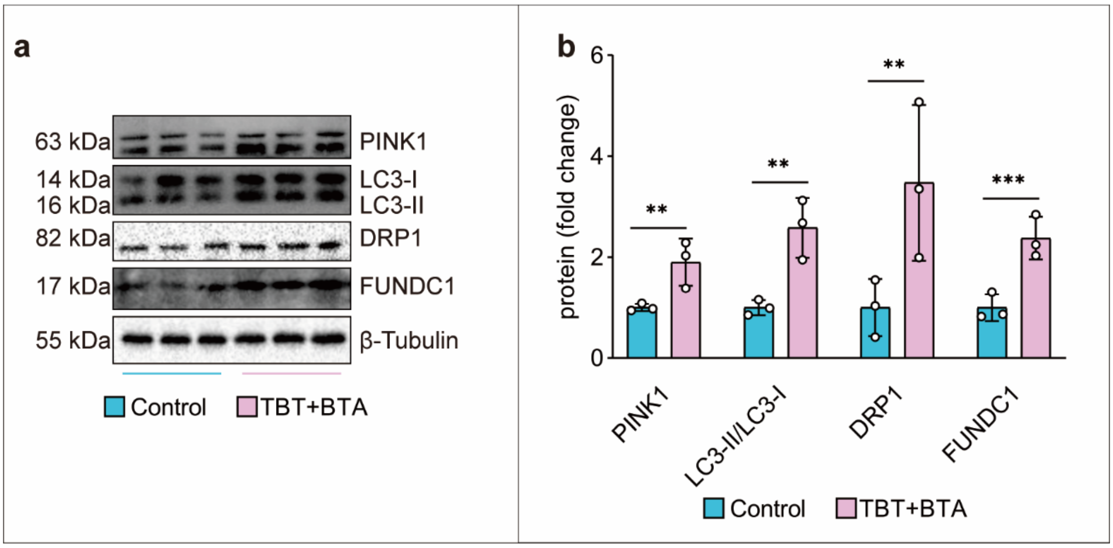

Supplementary Fig. 7. Inhibition of IDH3 and CIC induces excessive mitophagy and enhances the expression of DRP1 and FUNDC1. a-b. MLE12 cells treated with TBT and BTA simultaneously had increased mitophagy related-protein expression, including PINK1 and LC3II/LC3I, and enhanced expression of DRP1 and FUNDC1 (n=3). Data are shown as mean ± SD. ** $P < 0.01$ and *** $P < 0.001$.

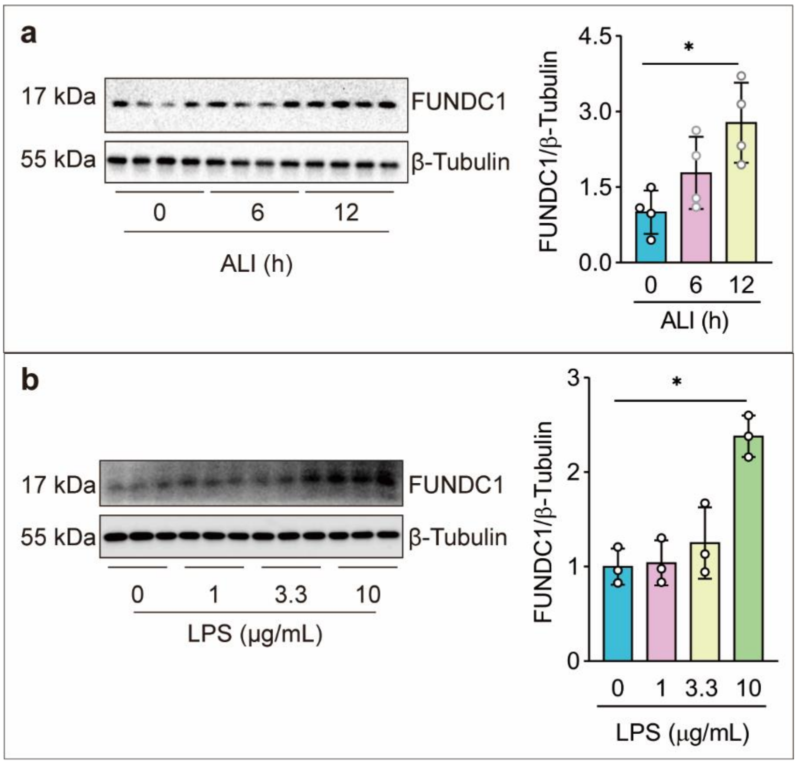

Supplementary Fig. 8. Elevated expression of FUNDC1 in the lungs and MLE12 cells with LPS challenge. a-b. The expression of FUNDC1 in the lungs (n=4) and MLE12 cells (n=3). Data are shown as mean ± SD. *$P < 0.05$.
